# EMT in Breast Carcinoma—A Review

**DOI:** 10.3390/jcm5070065

**Published:** 2016-07-14

**Authors:** Joema Felipe Lima, Sharon Nofech-Mozes, Jane Bayani, John M. S. Bartlett

**Affiliations:** 1 Ontario Institute for Cancer Research, Toronto, ON M5G 0A3, Canada; joema.lima@oicr.on.ca (J.F.L.); jane.bayani@oicr.on.ca (J.B.); 2 Sunnybrook Health Sciences Centre, Toronto, ON M4N 3M5, Canada; sharon.nofech-mozes@sunnybrook.ca

**Keywords:** EMT, breast, cancer, invasion, metastasis

## Abstract

The epithelial to mesenchymal transition (EMT) is a cellular program that is involved in embryonic development; wound healing, but also in tumorigenesis. Breast carcinoma (BC) is the most common cancer in women worldwide, and the majority of deaths (90%) are caused by invasion and metastasis. The EMT plays an important role in invasion and subsequent metastasis. Several distinct biological events integrate a cascade that leads not only to a change from an epithelial to mesenchymal phenotype, but allows for detachment, migration, invasion and ultimately, colonization of a second site. Understanding the biological intricacies of the EMT may provide important insights that lead to the development of therapeutic targets in pre-invasive and invasive breast cancer, and could be used as biomarkers identifying tumor subsets with greater chances of recurrence, metastasis and therapeutic resistance leading to death.

## 1. Introduction 

The epithelial to mesenchymal transition (EMT) is a complex program in which epithelial cells acquire a mesenchymal phenotype and motility through a cascade of biological events. There are three types of EMT programs; type 1 relates to embryogenesis, gastrulation and neural crest formation; type 2 is related to tissue regeneration and wound healing; and type 3 is associated with malignancy, invasion and metastasis [1,2,3]. The EMT is a natural phenomenon in development and wound repair but also represents one of the “hallmarks of cancer” [2]. During tumorigenesis, changes in EMT regulatory pathways lead to a loss of cellular adhesions, changes in the polarization of the cell and cytoskeleton, detachment, migration, intra-vasation, and survival in the vascular system; extravasation, and finally, metastasis [3,4]. Morphologically, the EMT is classically characterized by the dedifferentiation from an epithelial to mesenchymal phenotype, marked by the decrease expression of E-cadherin and increased expression of N-cadherin, vimentin as well as expression of cellular proteases. The EMT is also believed to be a critical step in the progression of cancers from both the pre-invasive to invasive state (e.g., in DCIS, PIN, etc.), and from organ confined to metastatic disease [4], though there is evidence to show that the EMT is not an obligate step to metastasis, as discussed further in this review. However, a pivotal challenge in the study of the EMT is the fact that it represents a transitory state. Studies suggest that in some situations, following the migration of a cancer cell which has undergone EMT to a distant site, a reverse process or mesenchymal to epithelial transition (MET) occurs. The MET is a state when a mesenchymal tumor cell reverts back to the epithelial phenotype, especially in distant metastatic sites [2]. 

**Figure 1:**
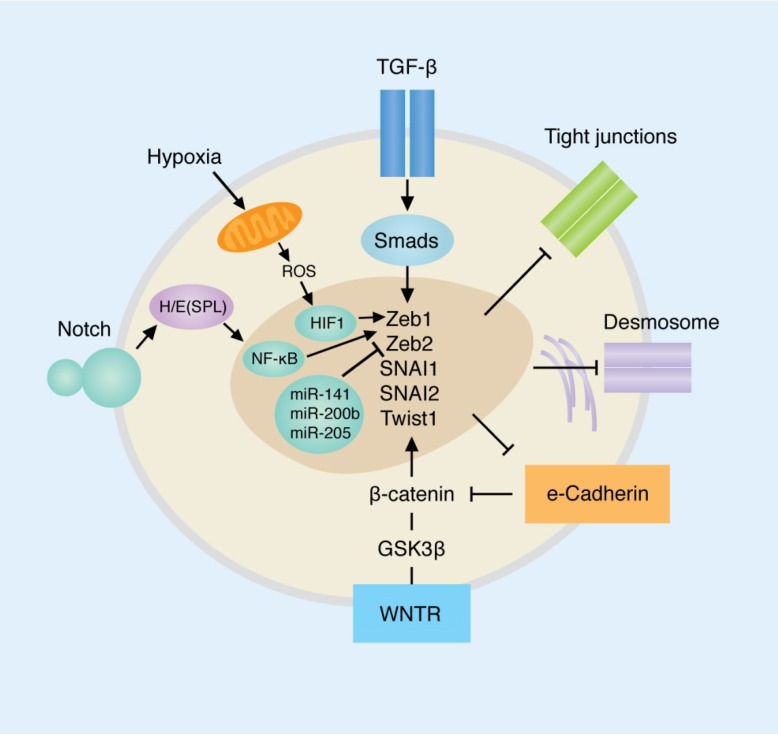
Epithelial to mesenchymal transition overview. Selected signaling pathways are depicted. Transforming growth factor-β, Notch, WNT can induce EMT through multiple pathways. EMT and MET (mesenchymal to epithelial transition) are associated with changes in the cytoskeleton and disruption of tight junctions and desmosomes. Adapted from Polyak et al. [5].

**Figure 2:**
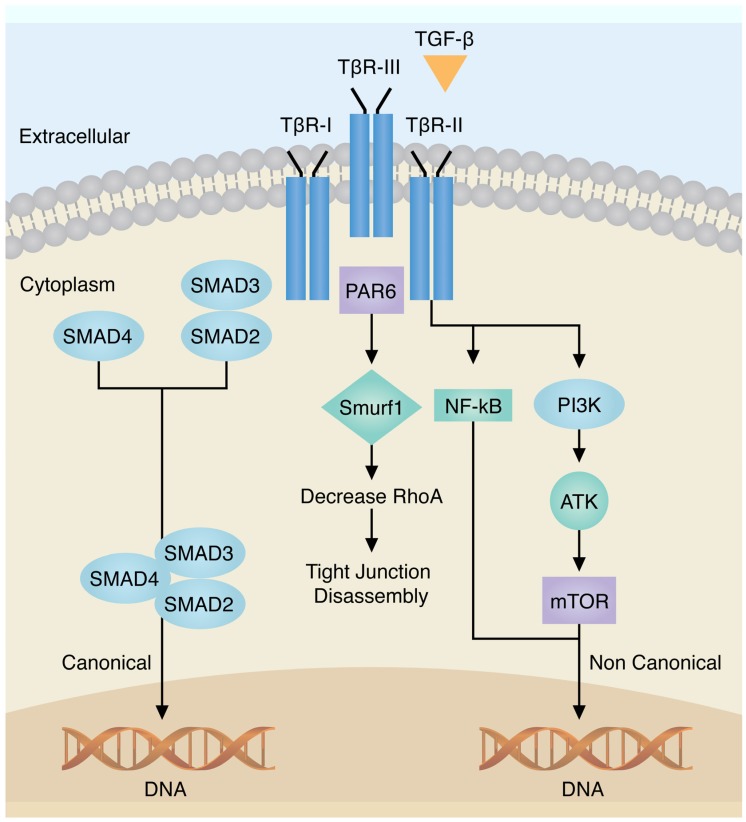
Transmembrane signaling by TGF-β. Canonical TGF binds to TβR-III, with recruitment of TβR-I, resulting in activation of Smad 3/2 and 4 translocate to the nucleus to regulate TGF-β genes (**left**); The non-canonical TGF binds to TβR-II (or III with subsequent binding of II) through Smad-independent pathway including Par-6, NF-kB, PI3K is shown (**right**). Adapted from Taylor et al. [6].

The master regulators of the EMT include many pathways, however the primary mediators of the EMT include signaling through TGF-β, Notch and Wnt; but are also influenced by the effects of the tumor microenvironment such as hypoxia as well as the differential expression of microRNAs (miRNAs) (Figure 1) [5,6]. Common to all these pathways are the convergence on the transcriptional factors SNAI (Snail), Zeb and Twist, whose differential expression in cancers has been shown to lead to the EMT. Indeed, both canonical and non-canonical TGF-β signaling [6] (Figure 2) lead to changes in the gene expression of the transcription factors Snail, Zeb, Twist and Six1. Similarly, Notch signaling acts on NF-κB, inducing the transcription of SNAI1, SNAI2 (Slug), Twist and Zeb1/Zeb2; as well as increasing cytokine production and cell survival. The Wnt pathway, often deregulated in cancer, leads to induction of SNAI1 expression with subsequent downregulation of E-cadherin via β-catenin. External stimulus, as in the case of hypoxia, leads to changes in mitochondrial function, leading to HIF1 stimulation, with subsequent Zeb1 expression. 

In this brief review, we will discuss the roles of these pathways in the EMT program as it pertains to its contribution to the progression of breast cancer and offer insight to the potential for biomarker discovery and therapeutic intervention.

## 2. Impact of the EMT on Breast Cancer

Both classical histological and molecular subtyping of breast cancers [1] have identified the impact of the EMT on breast cancer prognosis. Clinical-histologic studies of basal-like breast cancers show that they are among the most aggressive and deadly breast cancer subtypes, displaying a high metastatic ability associated with mesenchymal features [1,3,4]. The molecular mechanism underlying the maintenance of mesenchymal features in the basal-type cancers remains obscure. However, KRAS activation appears to be more prominent among this group, more so than in luminal tumors; implicating a role for KRAS in maintaining the mesenchymal phenotypes via SNAI2 expression [4]. Unsupervised hierarchical clustering performed by Sarrio et al. showed an up-regulation of the classical EMT markers (vimentin, smooth-muscle actin, N-cadherin and cadherin-11); overexpression of extracellular matrix proteins and invasion-associated genes (SPARC, laminin, and fascin), with reduction of epithelial markers among basal-like tumors [7]. 

Transcriptional profiling has also helped to identify an EMT gene-expression signature associated with claudin-low and metaplastic breast cancers; which correlates these tumors with a lower complete pathologic response and poor survival among these patients. These tumors were shown to display differential expression of Snail, Twist and TGF-β. However, the authors emphasize, that the expression of these markers is heterogeneous across the tumor, implicating a role for the stroma in the biology of the tumors, and the contribution of induction of the EMT by the surrounding tissues [8]. 

Since breast cancers are driven by the aberrant hormone-dependent pathways, the hypothesis that loss of estrogen receptor (ER) function results in trans-differentiation from an epithelial to a mesenchymal phenotype, with increased aggressiveness and metastatic potential, has been explored by several authors in recent years [9]. For example, the siRNA-mediated silencing of ER in cell lines like MCF7 resulted in cells with altered morphology, increased motility and switch from keratin/actin to a vimentin based cytoskeleton [9]. In this context, the various intrinsic subtypes of breast cancer have been receiving increased attention in stratifying breast cancer, especially in terms of prognostic and predictive information [10]. There is currently no extensive data on luminal breast carcinomas; however, immunohistochemistry-based platforms analyzing the expression of transcription factors, SLUG and SOX9, which are involved both in embryonic development, across 617 breast carcinomas by tissue microarray, showed the general loss of SOX9 expression in what was classified as luminal A and B, and moderate loss among those classed as triple negative [10]. Additionally, SLUG was expressed among of triple negative cancers, while not expressed among luminal tumors [10]. Although the classification of the tumors into subtypes was performed using ER, progesterone receptor (PgR) and HER2 immunohistochemistry, rather than by transcriptional profiling, the authors concluded that there was an inversed preferential nuclear expression of SLUG, SOX10 and SOX9 in triple negative tumors and non-triple negative tumors—that is, loss of SOX9 in luminal and HER2 positive cancers; moderate loss of SOX9 in triple negative cancers; loss of SLUG in luminal and HER2 positive cancers; expression of SLUG in triple negative cancers; expression of SOX10 in luminal and HER2 positive tumors, and lower expression of SOX10 in triple negative cancers [10].

These histologic and clinical observations speak to the biological heterogeneity of breast cancers [4]. Scimeca et al. investigated the correlation among poorly differentiated carcinomas and expression of NF-kB, Sonic Hedgehog (SHH), K-RAS, and PTX3 in 100 breast cancer biopsies. The different number of cells positive for vimentin, nuclear β-catenin, and CD44 expression, in high-grade ductal infiltrating carcinoma, as compared to low-grade carcinoma and benign lesions suggested that the process of de-differentiation of breast cancer cells could be related to the EMT [4].

Furthermore, molecular subtyping [1] supports the histological observations showing the so-called Luminal A/B and HER2-enriched cancers as those which retain a more epithelial phenotype; while the non-luminal cancers, comprising the triple negative or basal-like cancers are characterized by more mesenchymal features [1,10]. These observations can be generally correlated clinically to the findings that basal-like cancers are constitutively more invasive than their non-basal counterparts [9]. 

In the context of progression from relatively benign lesions to ductal carcinoma in situ (DCIS), preclinical data shows that understanding specific differences in gene expression is important to understanding progression to invasive disease [11]. Among the genes, those that have been implicated as key players in the EMT in DCIS and ADH include SNAI2, TGFβR3, EPCAM and SOX4 [12]. However, to date, much of the work investigating the role of the EMT in breast cancers has focused around the non-luminal subtypes and cancers in the metastatic setting. A recent gene transcriptional study demonstrated that gene expression signatures obtained from normal mammary stem cells are most similar to claudin-low profiles. This is consistent with findings by Taube et al.*,* showing that expression changes associated with the EMT correlates closely to claudin-low tumors and metaplastic carcinomas [2]. Micalizzi et al. highlighted the findings that triple-negative breast cancer displays up-regulation of EMT markers (vimentin, smooth-muscle-actin, N-cadherin, and cadherin-11) and overexpression of proteins involved in extracellular matrix remodeling and invasion (SPARC, laminin, and fascin); together with reduced expression of epithelial markers (E-cadherin and cytokeratins), which preferentially occur in breast tumors with the “basal-like phenotype”, linking EMT with the latter [3]. 

The role of the EMT among other breast cancer subtypes however has yet to be fully elucidated. Another relevant concept in the role of the EMT on breast pathogenesis is that the MET contributes significantly once invasion or metastasis has been accomplished. In this way, the mesenchymal tumor cell acquires or reverts back to its epithelial phenotype at the site of metastasis [3]. 

### EMT in the Progression to Metastatic Disease

There are numerous studies describing the role of the EMT in breast cancer progression and metastasis [3,13,14,15,16,17]. Transcriptional profiling, to elucidate differences between metaplastic carcinoma and invasive ductal carcinoma, identified the downregulation of epithelial genes, such as E-cadherin, and upregulation of genes related to the production of the extracellular matrix [8], and mesenchymal state hallmarks such fibronectin and N-cadherin. However, because the EMT reflects a transitional state, it is difficult to readily identify, in clinical samples, whether EMT/MET has occurred; therefore the contribution of the EMT in breast cancer progression has been evaluated primarily in cell line models. For example, in an ERα knockdown model of MCF-7 by Bouris et al., the demonstrated loss of ER expression was associated with the acquisition of the EMT phenotype, including enhance proliferation and migration, as well as changes among components of the extracellular matrix and matrix metalloproteases [15]. Additionally, Faronato et al. showed that DMXL2, a novel regulator of Notch signaling was overexpressed in a subset of breast carcinoma cell lines and similarly associated with features of the epithelial to mesenchymal transition [18].

Arguably, there is also data suggesting that disease progression and metastasis is mediated by other mechanisms aside from the EMT. Fischer et al. showed that breast cancer progression may occur in the absence of the EMT, by demonstrating that mammary tumors in mice can metastasize to the lung without going through an apparent EMT process. The resulting lung metastases consisted mainly of non-EMT tumor cells that maintained their epithelial phenotype as shown by the EMT lineage tracing experiments [19]. Moreover, Fischer et al. suggested that while modulation of EMT-associated transcription factors could be achieved, it does not necessarily impact on metastatic potential [19]. 

## 3. Primary Pathways Involved in EMT and Their Roles in Breast Cancer

### 3.1. Transforming Growth Factor-β

Transforming growth factor-β is a multifunctional cytokine involved in several physiological processes, including the EMT [6]. Mammals express three distinct isoforms of TGF-β: TGF-β1, TGF-β2 and TGF-β3 [6], which are associated with three different receptors: types I, II and III. TGF-βR-III is the most abundant receptor that works as an accessory receptor presenting the TGF-β to its signaling receptors. Binding of TGF-βs to TGF-βR-II enables the recruitment and activation of TGF-βR-I, leading to induction of canonical Smad 2/3-dependent signaling [3,6]. Both canonical and non-canonical TGF-β signaling systems are involved in EMT as shown in Figure 2. Specifically, TGF-β receptors activate Smad 2 and Smad 3, resulting in an activated complex composed of Smad 2/3 that further complexes with Smad 4 [6]. This complex interacts with several transcription factors to regulate genes involved in the EMT including the direct phosphorylation of receptors of SMAD transcription factors and cytoplasmic proteins regulating cell polarity. The overexpression of Smad 2 and Smad 3 also contributes to the EMT, while the reduction of their expression is associated with decreased metastatic potential [3,6] (see Figure 2). Non-canonical EMT TGFβ signaling is represented by several pathways including NF-κB, Par6, small GTPases, PI3K/AKT/TOR and MAP kinase family with activation of ZEB-1. These pathways regulate distinct processes including cytoskeleton organization, cell survival, migration and invasion [3,5,6]. Gene expression induced by TGF-β includes the transcription factors Snail (SNAI1 and SNAI2/Slug), ZEB, Twist and Six family of homeobox (Six1) [3].

Among breast cancers, TGF-β over-expression has been implicated in the EMT by mediating the functional conversion of TGF-β during breast cancer progression, suggesting that the chemotherapeutic targeting of EMT induced by TGF-β may offer new inroads in treating metastatic disease in breast cancer patients [3,13]. 

### 3.2. E-Cadherin

#### 3.2.1. E-Cadherin Loss in EMT Leads to Loss of Cell-Cell Adhesion

E-cadherin, a transmembrane glycoprotein, establishes homophilic interactions with adjacent E-cadherin molecules expressed by neighboring epithelial cells [20,21]. Expressed by the CDH1 gene, E-cadherin is a critical in the EMT. Upon downregulation, epithelial cells acquire a fibroblastic phenotype, enabling the dissociation of the epithelium and enhanced migratory capabilities [20]. This process is essential for the processes including gastrulation, neural crest formation, kidney development, etc. Silencing or reduction of CDH1 can occur either by somatic mutation, chromosomal deletions, proteolytic cleavage, and silencing of CDH1 promoter by epigenetic modification, ultimately resulting in the disruption of tight junctions and desmosomes [21]. In addition to increasing migration potential, E-cadherin downregulation also increases resistance to apoptosis [20,21]. 

Alterations in E-cadherin can occur genomically, transcriptional and post-transcriptionally, however, there is controversy regarding the impact of these different mechanisms of E-cadherin inactivation. The primary mechanisms of E-cadherin inactivation include mutation or promoter hypermethylation [20,21,22]. Lombartaes et al. observed that CDH1 promoter hypermethylation, but not CDH1 mutational inactivation, in the context of the EMT, resulted in a more aggressive tumor cell phenotype, and increased invasiveness [20]. However, this study had the limitation of analyzing expressions in cell lines instead of tissue samples [20]. In contrast, a recent study by Ciriello et al., presented a description of molecular portraits of invasive lobular breast cancer that does not support the occurrence of CDH1 epigenetic silencing in invasive lobular breast cancer [21]. Although there is controversy as to whether hypermethylation is the most common mechanism of CDH1 silencing associated with the EMT, and conferring more aggressive tumor behavior, it is known that epigenetic regulatory programs involve DNA methylation, chromatin (histone) modification, and non-coding RNA (small non-coding RNAs or micro-RNAs) in cancer stem cell associated with the EMT [3,21,22].

E-cadherin expression may also be repressed through the action of TGF-β, via its ability to induce the expression of Snail/ZEB family [3]. It has been shown that Snail may also form complexes that recruit different chromatin modifying enzymes and cofactors leading to DNA hypermethylation of the CDH1 promoter and silencing of E-cadherin expression [20,21,22]. Snail not only represses E-cadherin expression, but also down-regulates the expression of claudins, occludins, and mucin-1; and inducing invasive behavior [3].

Elevation of N-cadherin expression, a known mesenchymal marker, with the concomitant decrease of E-cadherin contributes to the loss of adhesion between tumor cells [22]; which is observed more prominently among basal-like breast cancers as well as in HER2+ tumors [18]. The clinically aggressive basal-like breast tumors show the highest intensity of N-cadherin expression, particularly close to vascular-rich areas [21,22]. The upregulation of N-cadherin in tumor cells that have diminished E-cadherin has been associated with elevated cell motility and poor clinical outcomes in patients [5,20,21,22]. 

#### 3.2.2. Migration and Invasion related to E-cadherin loss and changes in Metalloproteinases

Loss of cell polarity and cell-cell junctions, and the partial disassembly of epithelial tissues are changes associated with cancer progression to an invasive and metastatic state [5]. Rearrangement of structural proteins, via the relocation of actin and E-cadherin from tight junctions to the cytoskeleton, promotes formation of stress fibers that increase cell strength, flexibility and migration. These influence changes in cell morphology, phenotype and migratory ability, and therefore are subjects of analysis when defining the EMT program [20]. While tumor cells can become more motile, increased migratory and invasive potential is also mediated by changes in the extracellular matrix surrounding tumor cells [5,22]. 

The basement membrane provides a physical barrier which impedes tumor cell invasion [15,16,23,24]. The penetration of this barrier is a pivotal step in tumor progression. During the EMT, tumor cells increase the production of proteases with the capacity to degrade the surrounding basement membrane [15,17,21]. Increased abundance of the metalloproteinases MMP14 (MT1), MMP9 and ADAMTS1 have been identified in tumors and believed to contribute to proteolytic cascades leading to basement membrane destruction and tumor cell invasion [15,17,24]. 

Matrix metalloproteinases (MMPs) are endopeptidases which are notably known for facilitating the degradation of the proteins of the extracellular matrix. However, MMPs can also cleave cell surface receptors and participate in the processing of bioactive molecules. Metalloproteinases 2 and 9 are highly associated with invasion and metastasis in EMT programming [5,17]. These observations support the notion that as part of the genetic reprogramming during EMT, tumor cells switch to a pro-invasive/migratory phenotype an essential component of the metastatic program [23,24].

### 3.3. WNT/β-Catenin Pathway

The activation of the WNT/β-catenin pathway is particularly important for tumorigenesis in general, and forms part of the EMT and epithelial plasticity in normal development [23]. Activation of WNT/β-catenin induces SNAI1 expression with subsequent down regulation of e-cadherin and upregulation of vimentin [23,24]. Aberrant β-catenin expression, as determined by assessment of its subcellular localization, constitutes a surrogate marker of Wnt signaling pathway activation, and has been reported in a subset of breast cancers [25,26,27]. However, the association of β-catenin/Wnt pathway activation with clinical outcome and the mechanisms leading to its activation in breast cancers still remain a matter of controversy [27,28], with some studies showing that cumulative alterations in more than one Wnt antagonist and increased accumulation of β-catenin play an important role in the development of breast carcinoma while other studies show that alterations in the Wnt pathway are associated with the EMT programming, but β-catenin alone, is not sufficient to induce the EMT [2,28]. 

In studies of spindle lesions of the breast, aberrant β-catenin expression is often observed in metaplastic breast carcinomas, suggesting that Wnt pathway activation is found in at least a subset of breast cancers [27]. Moreover, β-catenin, also acts as a molecular bridge to enhance cell-cell adhesion in the tight junctions of epithelial cells, when coupled with E-cadherin [28]. In the absence of Wnt, cytoplasmic β-catenin is constantly degraded by the action of the Axin complex (Axin protein, APC gene product, casein kinase 1 and glycogen synthase kinase 3), with subsequent ubiquitination and proteasomal degradation [25,26,27]. β-catenin stabilization, following Wnt activation, results in its higher nuclear levels and pathological effects. One of these effects is the association with a transcription factor (TCF/LEF) and promotion of the expression of target genes involved in EMT [5]. 

### 3.4. Notch

Notch signaling normally balances cell proliferation, differentiation and apoptosis; activates the NF-κB pathway, and modulates TGF-β orchestration of EMT [6,29,30]. In normal mammary gland development, the Notch pathway is associated with the regulation of cell survival and has been implicated in cancer initiation and progression [29,30]. Recent studies have shown that Notch signaling is essential for maintaining the cancer stem cell-like population in a breast cancer cell line [29]. Notch signaling controls Snail expression by two synergistic mechanisms: including direct transcriptional activation of Snail, and an indirect mechanism operating via lysyl oxidase (LOX) [30]. Notch also increases LOX by recruiting HIF-1α, which stabilizes Snail, resulting in up-regulation of the EMT program, and subsequent migration and invasion of cancer cells. Meta-analysis of 3867 breast carcinomas showed that increased Notch expression has been associated with progression and poor prognosis, with higher Notch over-expression among the basal cancers [31]. Currently, Notch inhibition is an experimental treatment strategy for several cancers, including breast carcinoma [5,29,30,31]. 

### 3.5. Hypoxia

Hypoxia is one of the major physiological drivers responsible for the EMT (Figure 2), including the upregulation of hypoxia-inducible factor 1-α (HIF1α), hepatocyte growth factor (HGF), SNAI1 and TWIST1; activation of the Notch or NF-κB pathways, and induction of DNA hypomethylation [5,32,33]. Evidence has shown that the hypoxic microenvironment plays a key role in regulating breast cancer progression and metastasis [32], with elevated expression of hypoxia-associated genes associated with poor prognosis. Indeed, studies by Yang et al. showed that expression of the HIF-1α gene, a regulator of the hypoxic response, or a hypoxia meta-gene signature was associated with a poor outcome to endocrine treatment in ERα (+) breast cancer. Moreover, HIF-1α was able to contribute to endocrine therapy resistance to ERα (+) breast cancer cells [32,33]. 

In vitro studies on multiple human cancer cell lines show that low (3%) oxygen levels can induce the EMT phenotype, by inhibiting the activity of GSK3β, thereby sparing β-catenin from phosphorylation and subsequent destruction, and leading to its stabilization [5]. In other studies, the activation of Notch signaling was required for hypoxia-induced EMT by activating WNT/β-catenin signaling with the resulting stimulation of SNAI1 [5,32]. Hypoxia may also activate self-reinforcing positive-feedback loops that help to stabilize the mesenchymal state [5]. 

### 3.6. Tumor Necrosis Factor-Alpha (TNF-α)

TNF-α is a pivotal cytokine involved in several processes including inflammation, cellular homeostasis and tumor progression [34,35]. It promotes cancer invasion and angiogenesis associated with EMT programs, through the intersection of pathways that contribute to the repression of E-cadherin and activation of MMP9 [5]. Though unclear, the mechanism EMT induction by TNF-α suggests that the upregulation of Twist-1 may be involved in the process [35]. Upregulation of TNF-α has been shown to be associated with increase metastatic potential and invasiveness in breast cancer [34]. 

It has been shown that the EMT and cancer “stemness” properties, induced by TNF-α, are mediated by the upregulation Twist-1 [35]. The exposure to TNF-α, induced Twist-1 mRNA and protein expression in breast cancer cells, cell lines and mouse models, leading to the hypothesis that there is a signaling axis through which the tumor microenvironment and TNF-α elicit Twist-1 expression to promote cancer metastasis [35]. The authors suggest that targeting NF-κB-mediated Twist-1 upregulation may offer an effective therapeutic strategy for breast cancer treatment [35]. In renal cell carcinomas (RCC), TNF-α induced EMT of RCC cells by repressing E-cadherin, promoting invasiveness and activating MMP9 activity [34]. 

### 3.7. MicroRNAs

miRNAs are an evolutionarily conserved class of small noncoding RNAs that control gene expression by targeting mRNAs by binding to the 3′-untraslated region (3′UTR), leading to reduced translation of proteins, or degradation of the target mRNAs [36,37,38,39]. Dicer, also known as endoribonuclease Dicer or helicase with RNase motif is an enzyme encoded by the *DICER1* gene. Being part of the RNase III family, Dicer cleaves double-stranded RNA (dsRNA) and pre-microRNA (pre-miRNA) into short double-stranded RNA fragments called small interfering RNAs (siRNAs) and miRNAs, respectively [39]. During mammary gland development, miRNAs act as regulators of gene expression [39,40]. In general, the levels of most mature (processed) miRNAs are decreased in cancer versus normal tissue [36,37]. 

miRNA-200c has been shown to suppress the EMT, by targeting ZEB1/ZEB2, and maintaining E-cadherin expression [37,38,39]. It is a member of the tumor suppressive family of miRNAs including miRNA-200a, -200b, -200c, -141 and -429. Differential expression of the miRNA-200 family, for example in the upregulation of miR-200c, increased Dicer expression in two ERα-negative cell lines [36,37,38,39]. Similarly, in a study by Rhodes et al., the overexpression of miR-200b-3p, in a triple negative breast cancer cell line with no expression of miRNA-200 members, repressed the EMT, as demonstrated by the decrease in migratory capability and increased CDH1 expression [37]. This suggests that loss of miR-200 family members contributes to the EMT, as members of the miR-200 family of miRNAs can directly target repressors of CDH1, ZEB-1 and ZEB-2 [37]. 

Another miRNA, miRNA-300, was shown by Yu et al. [40] to be downregulated in breast cancer cells undergoing EMT, when compared to cells with a typical epithelial phenotype. miRNA-300 was shown to target Twist and negatively regulate the EMT and invasion [40].

Although the majority of miRNAs are decreased after neoplastic transformation, some miRNAs may be increased [39]. For example overexpression of miRNA-103/107 targets Dicer and inhibits its expression, thereby promoting EMT in breast cancer cell lines and metastasis in murine cell lines [39]. miR-10b, which represses homeobox D10 expression, releases transcriptional inhibition of the prometastatic Ras homolog gene family member C, while miRNA-221/222 can upregulate β-catenin expression and increased its the nuclear fraction [37]. 

Another miRNA involved in breast cancer metastasis and invasion in the context of the EMT is miR-10b. miR-10b is associated with mesenchymal features and invasive properties in breast cancer when overexpressed, through translational inhibition of HOXD10 (transcription factor associated with Wilms tumor) and upregulation of RHOC protein levels, enabling matrix extracellular degradation [5]. 

Finally, miRNA-21 has been identified to be overexpressed in several malignancies, and is known to promote breast cancer growth, proliferation, migration and metastasis partly by inhibiting PI3K/Akt targeting PIK3R1 and reversing EMT [5,39,40,41]. 

### 3.8. Cancer Stem Cell and EMT in Breast Cancer

Poor cancer survival has been linked to the enrichment of cancer stem cells that are capable of undergoing invasion, migration, survival, colonization and metastasis. Cancer stem cells are defined as those cells within a tumor that can self-renew and drive tumorigenesis, and are typically described as being CD44^+^/CD24^−^. The cancer stem-cell concept has important implications for cancer therapy [5,42]. In breast cancer, the properties of “stemness” is measured in vitro by the ability to form mammospheres, form tumors when injected into mouse models, and the ability to promote metastasis [5,42,43,44,45,46,47]. The cancer stem cell properties of breast cancer exhibit great phenotypical plasticity which allows the cell to undergo EMT at the site of primary tumor [42,44,45,46,47]. The expression of EMT-associated genes in CD44^+^/CD24^−^ cells isolated from normal human breast tissue, and from primary breast cancer, showed that many of the EMT transcription factors were expressed more so when cells had stem cell-like features [5,42]. 

In addition to its contribution to tumor progression and metastasis, the EMT has implications for therapeutic resistance. Moreover, the stem cell phenotype has also been associated with an increased resistance to apoptosis [46]. Highlighting the importance of EMT in therapeutic resistance, cancer cells with stem cell features have been found to be enriched in the residual tumors remaining after standard chemotherapy. In patients with breast cancer undergoing neoadjuvant therapy, there was an increase of CD44^+^/CD24^−^ cells expressing EMT-associated genes found in the post-treatment biopsy [46].

## 4. EMT Clinical Behavior, Tumor Invasion and Metastasis

Invasion is one of the central hallmarks of cancer. In this context, EMT as a complex reprogramming process of the epithelial cell is thought to play an indispensable role. Much of the evidence for an EMT role in invasion and metastasis is derived from experimental studies where the EMT can be induced in vitro [15,17,24,34,35]. Mesenchymal-like cancer cells invade tissues and migrate to distant sites through mechanisms similar to those observed in normal developmental embryogenesis [17,46,47,48]. 

Interestingly, the EMT rarely occurs homogenously across the whole tumor [13]. For invasion with subsequent metastasis, it is hypothesized that breast cancer tumor cells at the tumor margin lose epithelial properties and acquire features of mesenchymal cells [48]. The EMT has been suggested to be of prime importance for tissue and vessel invasion. For instance, when analyzing a sample with several components; in situ and invasive lesions, the expression of EMT markers will be differently expressed among different compartments of the cancer (DCIS, invasive breast cancer and, normal) in the same patient [48]. Alkatout et al. showed that SNAI1, SNAI2, Twist and Zeb1 are differentially expressed between normal breast epithelium, ductal carcinoma in situ and invasive breast cancer. Both invasive and in situ carcinoma expressed less Slug and Twist and more Zeb1 compared to normal epithelium. However, in lobular carcinomas [20], the expression of EMT markers appears uniformly throughout the cancer, in contrast to other tumors and breast carcinomas where the changes are observed only in subsets of cancer cells. Clearly, interactions between other tumor pathways and EMT may modulate invasion in different molecular and pathological subtypes. The transient nature of expression of EMT markers also complicates strategies for understanding and ultimately targeting these pathways [48]. 

## 5. Potential Prognostic and Predictive Value of EMT in Breast Cancer

EMT-gene signatures and biomarkers in BC and in other tumors may act as both prognostic (residual risk) and predictive assays [45]. The potential progression from invasive BC cancer to metastasis may better be understood by understanding EMT mechanisms leading to potential novel treatment decisions. In this respect, the EMT may provide opportunities for novel targeted therapies, and there are several drugs currently being tested for different tumors that are “anti-EMT” (discussed below).

To date, there are no commercial gene signatures that are prognostic or predictive that focuses specifically on the genes of the EMT. A current need is the elucidation of markers for ductal carcinoma in situ, where identification of tumors with the potential to recur; or to progress to locally invasive or metastatic disease, would significantly impact patient treatment with respect to radiotherapy or surgical resection alone. Along these lines, Knudsen et al., investigated the differences in gene expression between ductal carcinoma in situ (DCIS) and invasive breast cancer epithelia, with their findings showing that EMT-associated genes were consistently upregulated in invasive disease, relative to pure DCIS [45]. Similarly, in a meta-analysis of gene expression signatures defining EMT during cancer progression, Grӧger et al., identified 365 genes from 10 datasets. From this list, 130 genes were shown to be differentially expressed, with 67 identified as being upregulated, and the remaining 63 found to be downregulated in breast cancer cells. Investigation of the pathways associated with these differentially expressed genes showed that they were predominantly associated with adhesion, migration, development, cell differentiation, proliferation, angiogenesis, wound healing, metabolism and others [45]. 

## 6. EMT Targeted Therapy

With the EMT program influence by many pathways, so are the opportunities for therapeutic intervention [40,49,50,51,52,53,54]. As mentioned previously, miRNAs play an important role in the EMT processes. One prominent miRNA, miRNA-300, has been investigated in epithelial tumors for its contribution to the EMT [40]. miRNA-300 has been found to be downregulated in head and neck squamous cell carcinomas and breast cancer cells that had undergone the EMT. In luciferase and rescue assays, miRNA-300 has been shown to directly target Twist. Clinically, miR-300 expression was found to inversely correlate with Twist expression, and reduced miR-300 was associated with metastasis in patients’ specimens, implicating miR-300 as a possible candidate for cancer therapy [41,42]. Antagonism of miRNA-21, one of the first miRNAs described revealed reversion of the EMT with stem cell phenotypes in breast cancer cells via AKT and ERK1/2 pathways by targeting PTEN. This information may be useful for developing new treatments [37,40]. 

Studies by Yen et al., used xenograft tumors, to evaluate the antitumor effect of the anti-Notch 2/3 antibody, OMP-59R5. Immunohistochemistry, RNA microarray, real-time PCR, and in vivo serial transplantation assays found when used as a single agent, or in combination with other chemotherapeutics, efficacy was demonstrated in a broad spectrum of epithelial tumors, including breast, lung, ovarian, and pancreatic cancers [48], and is currently being explored [49,50,51].

Similarly, Song et al., found that Sinomemine, a drug used for rheumatic diseases, can induce apoptosis in several cell types by suppressing NF-κB. Co-immunoprecipitation analysis revealed that Sinomenine enhanced the binding of NF-κB and IκB in a dose-dependent manner, suggesting its effect on inactivation of NF-κB [52]. 

Targeting Wnt/β-catenin was demonstrated in a study using cell lines and xenografts, using the phytochemical, Baicalein, which downregulated the expression of Wnt1 and β-catenin proteins, as well as the and transcriptional level of Wnt/β-catenin-targeted genes. Baicalein was able to suppressed proliferation, migration, and invasion of MDA-MB-231 cells and on assays carried out in xenograft mouse model, inhibiting tumor metastasis in vivo [53]. 

Finally, the use of a pan-inhibitor of Aurora kinases, danusertib, promoted cellular apoptosis and autophagy, and inhibited the EMT in breast cancer cells, via modulation of p38 MAPK/Erk 1/2/Akt/mTOR signaling pathways, making this a promising anticancer treatment for breast cancer [49]. 

## 7. Conclusions 

The understanding of EMT programs in breast cancer has evolved in the last few years, from the recognition of the role for E-cadherin repression, to a complex molecular integration of multiple pathways. During the EMT, tumor cells acquire invasive traits through overexpression, mutation or amplification of oncogenes and also repression of tumor suppressors, leading to the aberrant expression of signaling pathways. In this brief overview of the EMT, it is clear that understanding the complex mechanisms underlying the change of the epithelial cell phenotype to a mesenchymal, and a more motile state, is critical to understanding breast cancer pathogenesis, and holds promise in providing predictive and prognostic biomarkers as well as novel therapeutic interventions.
